# Macromolecule-Assisted *de novo* Protein Folding

**DOI:** 10.3390/ijms130810368

**Published:** 2012-08-20

**Authors:** Seong Il Choi, Ahyun Son, Keo-Heun Lim, Hotcherl Jeong, Baik L. Seong

**Affiliations:** 1 Translational Research Center for Protein Function Control, Yonsei University, Seoul 120-749, Korea; 2 Department of Biotechnology, College of Bioscience and Biotechnology, Yonsei University, Seoul 120-749, Korea; E-Mails: 50hyuny@naver.com (A.S.); keo-heun@hanmail.net (K.-H.L.); 3 Vismer Co., Ltd., Ansan, Kyeonggi-do 426-791, Korea

**Keywords:** folding, macromolecules, aggregation, hydrophobic interaction, molecular chaperone, surface charges, excluded volume

## Abstract

In the processes of protein synthesis and folding, newly synthesized polypeptides are tightly connected to the macromolecules, such as ribosomes, lipid bilayers, or cotranslationally folded domains in multidomain proteins, representing a hallmark of *de novo* protein folding environments *in vivo*. Such linkage effects on the aggregation of endogenous polypeptides have been largely neglected, although all these macromolecules have been known to effectively and robustly solubilize their linked heterologous proteins in fusion or display technology. Thus, their roles in the aggregation of linked endogenous polypeptides need to be elucidated and incorporated into the mechanisms of *de novo* protein folding *in vivo*. In the classic hydrophobic interaction-based stabilizing mechanism underlying the molecular chaperone-assisted protein folding, it has been assumed that the macromolecules connected through a simple linkage without hydrophobic interactions and conformational changes would make no effect on the aggregation of their linked polypeptide chains. However, an increasing line of evidence indicates that the intrinsic properties of soluble macromolecules, especially their surface charges and excluded volume, could be important and universal factors for stabilizing their linked polypeptides against aggregation. Taken together, these macromolecules could act as folding helpers by keeping their linked nascent chains in a folding-competent state. The folding assistance provided by these macromolecules in the linkage context would give new insights into *de novo* protein folding inside the cell.

## 1. Introduction

What kinds of factors would drive newly synthesized polypeptides to efficiently fold into their native structures in crowded cellular environments? Over the last two decades, this question has been posed among other major issues in biology [1–4]. Despite much progress in our understanding of the “assisted” *de novo* protein folding *in vivo*, it still remains largely unsolved. Of note, it is extremely difficult to unambiguously uncover the underlying principles of the processes that include folding, misfolding, and aggregation due to the following reasons: They contain many complex features, such as transient and heterogeneous conformations, strict dependence of biological functions and physicochemical properties of proteins on their conformational states, and the irreversibility and multiphasic behavior of aggregation. In particular, elucidating the mechanisms of such processes *in vivo* becomes almost enigmatic when the influences of the cellular milieu are further taken into account. Along with these features, the diversity and flexibility of proteins would render the currently accepted fundamental principles fragile and biased with many exceptions and neglected aspects.

Efforts have been made to establish a single unifying principle governing protein folding. Indeed, the advent of new paradigms, such as the Anfinsen postulate and the concept of molecular chaperone, revolutionized our understanding of protein folding *in vitro* and *in vivo* [1,5]. Nonetheless, as the aforementioned complexity of the protein world implies, they appear to lack some important aspects, calling for a new viewpoint for describing *de novo* protein folding *in vivo*. In this review, we reexamine the Anfinsen postulate and the current status of molecular chaperones. We then discuss the folding assistance that is mediated by the nascent chain-linked macromolecules and their stabilizing factors against aggregation.

## 2. Uncovered Aspects of the Anfinsen Postulate and Molecular Chaperones

### 2.1. Anfinsen Postulate

Using a simple *in vitro* refolding system, Christian Anfinsen showed that all the information required for the native structure of a protein is encoded in its amino acid sequence [5]. According to his postulate, proteins can fold spontaneously, and their native structures are thermodynamically the most stable. This led us to a misconception that protein folding *in vivo* would occur in the same manner as the refolding *in vitro*. This principle turned out to be insufficient for explaining protein folding *in vivo*, based on the following considerations.

First, the Gibbs free energy of intramolecular folding conceptually provides no direct information about intermolecular association among polypeptides leading to aggregation, as pointed out previously [6]. Second, whereas single-domain proteins can fold *in vitro* in the time scale of micro-seconds to seconds, the refolding rates of larger multidomain proteins are sometimes very slow in the range of several hours to a day [7]. Such a long time scale *in vitro* appears to be physiologically irrelevant. In contrast, cotranslational folding (at least domain-wise cotranslational folding) facilitates multidomain proteins to fold rapidly [8–11] and thus can play a decisive role in *de novo* protein folding. Moreover, translational pausing at rare codons was reported to significantly affect folding yields and even final conformations [12,13]. In addition, there exist folding catalysts, such as peptidyl prolyl isomerase and protein disulfide isomerase [14,15]. These cotranslational folding events and folding catalysts strongly indicate that protein folding can be pathway-dependent or under kinetic control. Finally, native structures are not necessarily minimum energy conformers as long as proteins can maintain their solubility by intrinsic or extrinsic factors. For example, a substantial fraction of proteins are intrinsically denatured proteins (IDPs) [16,17]. IDPs alone can maintain their solubility under physiological conditions due to relatively higher net charge [18]. The model of conformational selection and population shift suggests that the sparsely populated pre-existing native conformers in the ensemble of IDPs can recognize and bind to their partners, subsequently leading to the population shift toward the native conformers [19]. In these cases, the native conformers are thermodynamically unstable and yet able to efficiently exert their biological functions. Given the widely distributed IDPs in the proteome, a plethora of proteins are likely incompatible with the Anfinsen’s thermodynamic hypothesis.

### 2.2. Molecular Chaperones

The principles of molecular chaperones have been gleaned mainly from the *in vitro* refolding experiments, using representative model proteins and simplified buffer solutions [2,3,20]. Obviously, however, there are significant differences between *in vitro* and *in vivo* environments with respect to cotranslational folding and macromolecular crowding [8–11,21]. Especially, the *in vitro* data of larger multidomain proteins regarding their folding rates and chaperone-dependent folding should be interpreted with every caution. For example, the *in vitro* refolding of firefly luciferase has been well known to be strictly dependent on the DnaK (*E. coli* hsp70 homolog) system [22,23]. However, *de novo* folding of firefly luciferase *in vivo* is independent of the DnaK system, although aggregation prevention and refolding of the denatured firefly luciferase during heat shock are dependent on this chaperone system [24].

A biochemical approach using the co-immunoprecipitation (co-IP) assay showed that trigger factor (TF), DnaK, and GroEL/GroES interact with 60%, 9%–18%, and 10% of newly synthesized polypeptides, respectively [3,25,26]. These findings led to the conclusion that *de novo* folding of these proteins *in vivo* is dependent on their interacting molecular chaperones [3,26]. In fact, it is this co-IP assay that decisively established the molecular chaperones as general folding helpers *in vivo*. However, it should be noted that the hydrophobic interactions, which are considered responsible for the substrate recognition of molecular chaperones, are involved in the inhibition of protein folding. Indeed, this inhibitory ability of molecular chaperones is crucial for protein translocation [27]. In addition, molecular chaperones interact with proteins to fulfill other cellular functions, including proteolysis, translocation, and signal transduction [28–30]. These functions are conceptually different from *de novo* protein folding. Frydman articulated the limit of the co-IP approach by pointing out “*Unlike the genetic approach, these studies do not establish that folding of a given substrate requires a given chaperone but only that an interaction occurs in vivo*.” [31].

Genetic approaches are crucial for revealing the role of molecular chaperones in protein folding *in vivo*. The single deletion of either *dnaK* or *tf* gene in *E. coli* strain turns out to make no detectable effect on protein folding, whereas the deletion of both genes increases protein aggregation [25]. This aggregation can be overcome by overexpressing GroEL/GroES or SecB [32,33]. Most notably, Masters *et al*. showed that the physical depletion (knock-down) of GroEL via a tightly controlled expression system has little or no effect on the folding of newly made proteins in *E. coli* [34]. They further suggested that an *E. coli* strain without GroEL could be constructed although the folding of several essential proteins depends on this chaperonin [34]. Furthermore, GroEL is either non-essential or absent at least in some mycoplasmas [35]. In contrast, a significant or wholesale protein aggregation was observed in the *E. coli* strains harboring the GroEL conditional mutant (E461K) at the non-permissive temperature [36,37]. It has been widely believed that protein aggregation at the non-permissive temperature resulted from a loss-of-function mutation in GroEL. But Masters *et al*. pointed out that this wholesale aggregation could result from a gain-of-function mutation in GroEL [34]. Consistent with this suggestion, the GroEL mutant (E461K) does not release its bound substrate due to the loss of proper allosteric communications at non-permissive temperature [37–39]. A timely release of substrate proteins from molecular chaperones is crucial for proper folding. Otherwise they can act as folding inhibitors. Likewise, a conditional mutant of the mitochondrial chaperonin, which resulted in the global aggregation of imported proteins in the yeast mitochondria, was observed to almost completely aggregate at the non-permissive temperature [40]. It is therefore of great necessity to distinguish the protein aggregation generated by a gain-of-function mutation in chaperonin from that done by a loss-of-function mutation in chaperonin.

## 3. Macromolecule Linkage-Mediated Folding Helper Systems *in Vivo*

**Figure 1:**
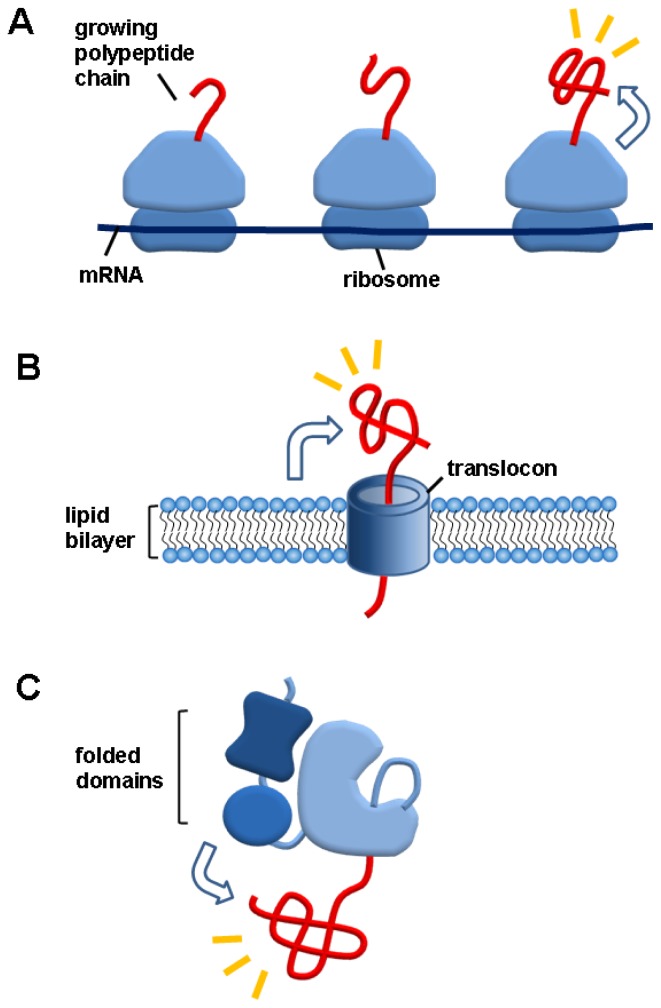
Macromolecule linkage-mediated folding helper systems *in vivo*. A hallmark of *de novo* folding environments *in vivo* is that newly synthesized polypeptides (red tube) are tightly connected to the macromolecules, such as ribosomes (top), lipid bilayers (middle), or cotranslationally folded domains in multidomain proteins (bottom). Although the linkage effects (represented by arrows) on the folding or aggregation of their linked endogenous polypeptides remain largely unknown, these macromolecules have been known to effectively and robustly solubilize their linked heterologous proteins. Thus, this known folding assistance of these macromolecules in the linkage context could be applied to *de novo* folding of endogenous proteins *in vivo*.

A hallmark of *de novo* protein folding environments *in vivo* is that, as illustrated in Figure 1, all nascent polypeptides without any exception are tightly connected to gigantic ribosomes (top), lipid bilayers in the case of secreted proteins across membranes (middle), or cotranslationally folded domains in the case of multidomain proteins (bottom). In particular, nascent chains are not released from ribosomes and lipid bilayers until the completion of translation and translocation, respectively. The potential aggregation problem of unfolded nascent chains on ribosomes or lipid bilayers has been a subject of intensive discussion in terms of *de novo* protein folding [3,41,42]. Moreover, the elucidation of the role of cotranslationally folded domains in their linked domains is crucial for understanding the *in vivo* folding of multidomain proteins. Nonetheless, the linkage effects of these macromolecules on the aggregation of their linked nascent chains have not been considered in the molecular chaperone-assisted *de novo* protein folding. By contrast, all these macromolecules have been known to solubilize their linked aggregation-prone heterologous proteins in the fusion technology or ribosome (or membrane surface) display technology [43–47]. In this review, we therefore suggest that the robust folding assistance of these macromolecules for their linked heterologous proteins could also play a pivotal role in *de novo* folding of “endogenous” proteins inside the cell.

### 3.1. Ribosomes

In 1986, Pelham put the following speculations on the roles of the hsc70 [48], contributing to the birth of the concept of molecular chaperone: “*What else might hsc70 do in unstressed cells? One possibility is that it recognizes nascent proteins, which by definition are “denatured,” and sorts out any aggregation problems that occur during their folding and assembly into oligomeric structures*.” The cooperative folding of domains and small proteins and the relatively slow rate of protein synthesis could potentially render the cytosol-exposed unfolded nascent chains on ribosomes as aggregation-prone for a significant timescale [3,41,42]. The degree of aggregation could be further increased by the high local effective concentration of nascent chains on the same polysomes and the macromolecular crowding inside the cell [3,21]. Importantly, these assumptions provided a rationale for the existence of the ribosome-tethered molecular chaperones such as TF in *E. coli* [49,50]. However, the deletion of the TF gene or eukaryotic counterparts has no effect on protein folding [25,51].

Ribosomes are megadalton-sized macromolecules (~2.4 MDa in *E. coli*) with polyanionic surfaces. It is questionable if the aggregation behaviors of nascent chains alone are similar to those of the same chains that are linked to ribosomes. Of note, hydrophobic interactions do not reflect the properties of non-contact regions, such as their size, shape and surfaces charges. Thus, in the hydrophobic interaction-dominated stabilizing mechanism, ribosomes seem to be unnecessary for the description of the aggregation of their linked nascent chains. In contrast, the surface charges (generating electrostatic repulsions) and excluded volume (generating steric hindrance) of macromolecules, including ribosomes and molecular chaperones, could serve as important stabilizing factors against the aggregation of their linked polypeptides [6,52]. These two factors are discussed later in more detail.

The linkage effects of ribosomes could be inferred from the study on a polyhistidine tagged luciferase immobilized on chelating Sepharose beads [53]. The inhibition of aggregation and subsequent increase in the refolding yield by the bead immobilization enabled the authors to suggest that like the beads, ribosomes as massive particles could carry out similar functions for their linked polypeptides [53]. Previously, we proposed that ribosomes could keep their linked polypeptides in an aggregation-resistant and folding-competent state, due to their surface charges and excluded volume [6,52,54]. Ribosomes can efficiently promote the solubility and folding of their surface-linked aggregation-prone proteins *in vivo* that are fused to the ribosomal protein [43]. The ribosome display technology has been proven useful for the production of highly aggregation-prone domains as a functionally active form [44]. Furthermore, the folding experiments of a ribosome surface-exposed polypeptide by ribosome stalling with optical tweezers showed that the bound polypeptide remains aggregation-resistant and folding-competent [55]. Taken together, the aggregation problem of nascent chains of endogenous proteins on the ribosomes should be understood with taking these known roles of ribosomes into account. To our knowledge, there has been no report showing that the nascent chains linked to ribosomes give rise to aggregation problem.

Ribosomes and their components including 50S subunit, 23S rRNA, and the domain V of 23S rRNA have been known to function as molecular chaperones *in vitro* by reversibly interacting with proteins [56,57]. The regions including peptidyl transferase center (PTC) in the domain V are responsible for their substrate recognition. Interestingly, either neutral or positively charged amino acids on the surfaces of native structures of the tested proteins are recognized by the domain V [58]. So far, it is unknown what stabilizing factors of ribosomes and its components maintain their bound substrate in a folding-competent state. Both the charge and steric factors of ribosomes and their components might be responsible for the stabilization of their bound proteins.

### 3.2. Lipid Bilayers

As is the case of ribosomes, the aggregation problem may also persist during the translocation of newly made polypeptides across lipid bilayers of membranes of endoplasmic reticulum, mitochondria, chloroplast, and cell. The linkage effects of membranes on the aggregation of translocating polypeptides have not been considered in most studies of *de novo* protein folding. The cell surface display technology allows protein of interest on the membranes or cell surfaces by fusing them to surface anchoring motifs [45,46,59–61]. The popular anchoring motifs for the *E. coli* outer membrane display include porins, lipoproteins, glycosyl-phophatidylinositol anchor, and β-autotransporters [60]. Importantly, the display of proteins to the cell (or membrane) surfaces greatly stabilizes their linked proteins [45,46,62]. Similarly, the immobilization of proteins on the surfaces of beads is known to be a powerful method for stabilizing proteins [63,64]. The cells or membranes that display proteins were thought to be equivalent to the beads that immobilize proteins [45]. Artificial systems they might be, the accumulating evidence supports that the membrane linkage could stabilize the aqueous phase-exposed polypeptides of endogenous secreted proteins or membrane-anchored proteins against aggregation.

The membrane linkage could affect the substrate-stabilizing ability of the membrane-anchored chaperones and components, such as calnexin and translocon: these molecules can be considered to be the components of membranes. In consistent with this idea, the chaperoning activity of the membrane-anchored calnexin is more effective than that of the ER lumen-exposed domain of calnexin in solution without membrane anchor [65]. In addition, when immobilized at the surface of solid matrix, a small heat shock protein, αB-crystallin, dramatically inhibit the aggregation of substrate proteins compared to αB-crystallin in solution without the immobilization [66].

### 3.3. Folded Domains in Multidomain Proteins

As evident in the *in vitro* refolding experiments with multidomain proteins, domains generally tend to inhibit the folding of other domains [7,67]. These *in vitro* experiments gave rise to the suggestion that multidomain proteins fold slowly and are aggregation-prone *in vivo*, thus necessitating assistance of molecular chaperones [3]. As mentioned before, the domain-wise cotranslational folding facilitates rapid folding of multidomain proteins *in vivo*, and yet the role of cotranslationally or independently folded domains remains largely unknown. In fact, demonstrating the linkage effects of folded domains, ribosomes, and membranes via the classic *in vitro* refolding method is not easy. It is partly due to the difficulties inherent to this method, where the denaturants required to unfold the target domains or linked proteins also impair or destroy the native forms of these macromolecules. In contrast, however, the *in vivo* fusion of aggregation-prone proteins to these macromolecules through genetic manipulation is relatively straightforward.

Fusion of soluble carriers to the *N*-termini of aggregation-prone proteins has been exploited as one of the efficient tools for overcoming aggregation of heterologous proteins in *E. coli* [68,69]. In essence, the artificial fusion proteins can be viewed as multidomain proteins, in which the *N*-terminal domains act as robust helpers for promoting the solubility and folding of their linked downstream domains. To demonstrate this chaperone-like function of *N*-terminal domains in natural multidomain proteins of *E. coli*, three *N*-terminal domains of *E. coli* proteins (lysyl tRNA synthetase, threonyl tRNA synthetase, and aconitase) were fused to the *N*-termini of highly aggregation-prone proteins [47]. All these domains profoundly enhanced the solubility and folding of a variety of their *C*-terminal heterologous proteins in the *E. coli* cytosol. Importantly, these findings strongly suggest that these *N*-terminal domains could also provide a similar chaperoning function for their authentic *C*-terminal domains *in vivo*, contributing to the autonomous folding of endogenous multidomain proteins. However, if the upstream domains fail to fold properly, they would interfere with the folding of downstream domains. Therefore, depending on their folding status, domains can act as either folding enhancers or inhibitors.

**Figure 2:**
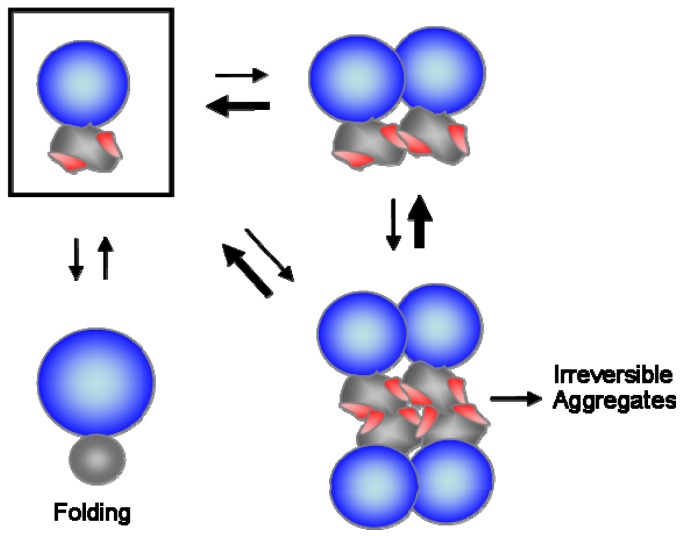
A model for how soluble folded *N*-terminal domains can increase the solubility and folding of their linked domains. The blue, wrinkled, and gray spheres represent the folded *N*-terminal domains, incompletely folded and folded *C*-terminal domains, respectively. The red spots on wrinkled sphere indicate the aggregation-prone regions. The electrostatic repulsions and steric hindrance of the folded *N*-terminal domains inhibit intermolecular association and shift the populations from the oligomeric state to the monomeric state (boxed), thus increasing the chance for the proper folding of *C*-terminal domains. Reproduced from [47].

The solubilizing ability of the *N*-terminal domains was shown to be closely correlated with their net charge and size. On the basis of these findings, a model was proposed to explain how soluble folded domains could increase the solubility and folding of their linked domains [47]. As shown in Figure 2, both electrostatic repulsions and steric hindrance of folded domains inhibit the intermolecular association and shift the populations toward the monomeric state, thus keeping the *C*-terminal domains in a folding-competent state. In particular, this model does not include the effects of interdomain interactions that could greatly influence the folding of multidomain proteins. Nevertheless, it explains well why soluble folded domains including soluble carriers can generally increase the solubility of a variety of their linked proteins. Importantly, this folding helper system mediated by soluble folded domains might be ubiquitous in the folding of endogenous multidomain proteins, because their charge and steric factors could stabilize their linked domains even without specific interdomain interactions.

### 3.4. Salient Features of the Macromolecule Linkage-Mediated Folding Helper Systems

The macromolecule linkage-mediated folding helper systems described in this review have several salient features for *de novo* protein folding. First, they exert the chaperoning function in the linkage context without ATP consumption, in much the same way as an intramolecular (or autocatalytic) reaction. Second, while linked to the macromolecules, proteins can fold, leading to cotranslational folding or rapid folding. Rapid folding would allow proteins to escape from the adverse aggregation enhanced by the macromolecular crowding effects [70]. This feature is distinct from the hydrophobic interaction-mediated mechanism by which protein folding generally stalls in the chaperone-bound state [27]. Third, soluble folded domains can persistently stabilize their linked domains against aggregation, basically independent of native interdomain interactions. Fourth, these systems can be conceptually applied to all nascent chains, as they are tightly linked to the ribosomes, lipid bilayers, or folded domains, throughout their biogenesis and folding. Lastly, nascent chains should communicate diligently with a variety of other molecules upon exposure to the cytosol. The interacting molecules include modifying enzymes, translocation systems, binding partners, and proteolytic systems [71,72]. These folding helper systems allow the nascent chains to fold in open space for the communication with their surroundings.

## 4. Hydrophobic Interaction-Based Stabilizing Mechanisms of Molecular Chaperones

Depending on what stabilizing factors of macromolecules including molecular chaperones are important, our understanding of assisted *de novo* protein folding could be profoundly influenced. Thus, it is necessary to discuss the possible factors of macromolecules involved in protein aggregation. In the energetics of protein structure and folding and the theoretical force fields for their simulations, various components are involved, including conformational changes, hydrophobic effect, hydrogen bond, electrostatic interaction, peptide solvation, backbone conformational entropy, surface charge, and excluded volume [73–76]. These components could be applied and extended to intermolecular aggregation. Consistently, the hydrophobic interactions and hydrogen bonds are known to be important for protein aggregation [3,77,78]. Molecular chaperones prevent misfolding and aggregation by transiently binding to the exposed hydrophobic surfaces of non-native structures [2,3]. The hydrophobic interaction-mediated substrate recognition and stabilization against aggregation underlie the prevailing working mechanisms of molecular chaperones [2,3]. However, it remains largely unknown whether the hydrophobic interactions are prerequisite for the substrate recognition or stabilization against aggregation. Other stabilizing mechanisms cannot be excluded. For instance, even molecular chaperones, including GroEL, hsp70, TF, calnexin, and calrecticulin, were reported to recognize their substrates mainly through the non-hydrophobic interactions, such as electrostatic interactions and glycan-binding [79–82].

GroEL assists protein folding by preventing aggregation without increasing folding rate (a passive but major role) or by increasing folding rate (an active role) [3,83–88]. Of note, the conformational changes of substrate protein by the bound chaperonin are not mandatory for the passive role of chaperonin [86]. This means that in the passive role of chaperonin, overcoming kinetic traps during folding process is entirely dependent on protein’s own intrinsic ability, even though the overall folding yield is strictly dependent on chaperonin. As the representative models of the active role of chaperonin, there are iterative annealing mechanism by which the chaperonin stimulates folding by repetitive unfolding and release, and the active cage model by which the confinement and interactions with cavity wall result in smoothing of protein’s energy landscape [3,85–88].

## 5. Charge and Steric Hindrance as Stabilizing Factors

### 5.1. Evidence for Charge and Steric Hindrance as Stabilizing Factors

Charge is a universal key factor determining the solubility of proteins as well as other molecules in the aqueous phase [89]. The net charge has been well established to be closely correlated with protein solubility [18,89–95]. For example, the supercharged variants of green fluorescence protein are extremely resistant to aggregation, such that they remain soluble even after boiling [94]. The aggregation rate is inversely correlated with the net charge [95]. The flanking charged residues or large soluble carriers, when covalently added, can increase the solubility of their linked polypeptides [96–99]. The net charge of soluble carriers is closely correlated with their solubilizing ability [47]. The increased electrostatic repulsions and hydration by charged groups are likely responsible for protein solubility. Taken together, accumulating evidence of the charge-mediated solubility enhancement clearly shows that the hydrophobic interaction-mediated direct contact is not a sole means for stabilizing the exposed hydrophobic regions against aggregation in the aqueous phase.

Protein aggregation is a specific process [100–102]. This means that sterospecificity is important for aggregation [103]. Thus, any steric hindrance resulting from the excluded volume of macromolecules, either covalently or non-covalently connected to proteins, is expected to profoundly inhibit aggregation. Previously, the steric hindrance of the macromolecules such as polysaccharides in *Peniophora lycii* phytase and polyethyleneglycol conjugated to human granulocyte colony stimulating factor was suggested to play a role in the stabilization of their linked proteins against aggregation [104,105]. The GroEL sequesters its substrate protein in the chamber, referred to as the Anfinsen cage, where an intermolecular interaction among substrate proteins is completely blocked [83]. In fact, this physical encapsulation can be viewed as a specific type of steric hindrance. In contrast, steric hindrance of macromolecules we here focus on can stabilize their linked polypeptides fully exposed to their surroundings.

### 5.2. Unique Features of the Charge and Steric Factors

**Figure 3:**
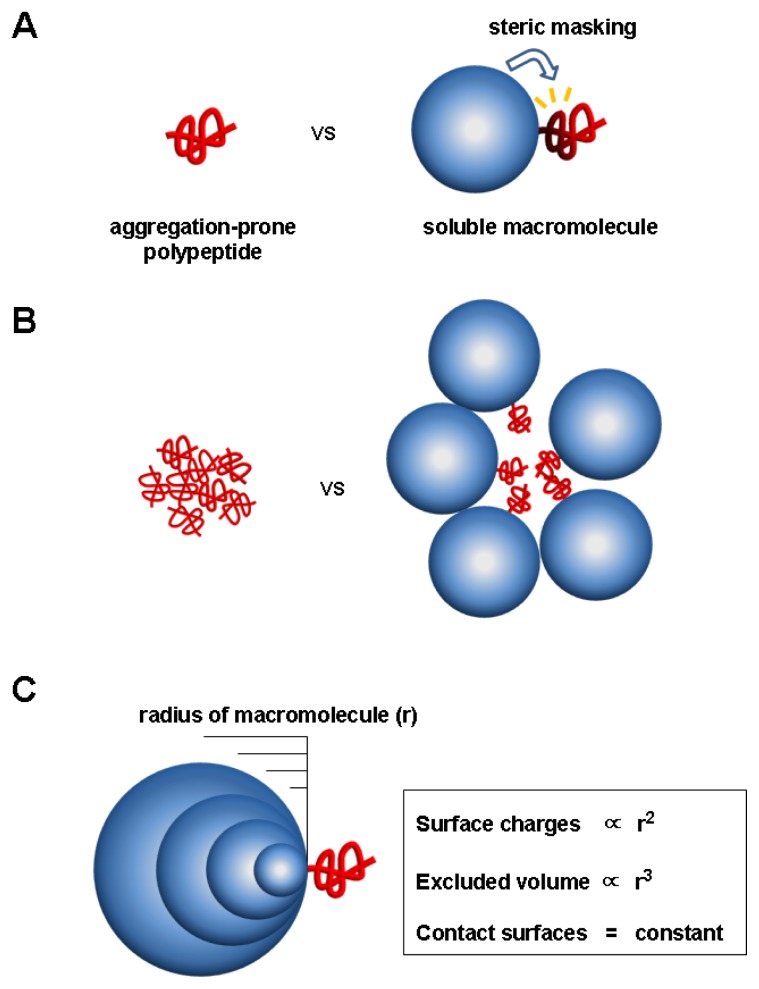
A simple model for assessing the stabilizing effects of the surface charges and excluded volume of macromolecule on the aggregation of its linked polypeptide. In this model, soluble macromolecule (blue sphere) with a constant surface charge density is linked to aggregation-prone polypeptide (red tube). (**A**) Unimolecular system. Assuming that macromolecule is covalently linked to the polypeptide without any conformational changes and intermolecular interactions between them, protein folding can be little affected by the macromolecule. But, as for aggregation, the local surfaces of polypeptide in close proximity to the macromolecule can be protected from intermolecular association by the direct steric masking of macromolecule. (**B**) Multimolecular system. In contrast to a unimolecular system, the total excluded volume and surfaces charges of the macromolecule can directly or indirectly inhibit the aggregation of the whole regions of the linked polypeptide in a multimolecular association. (**C**) Correlation of the stabilizing factors (surface charges, excluded volume, hydrophobic interactions) of macromolecule with its size. The soluble macromolecule with varying radius (*r*) is linked to a polypeptide through limited and constant hydrophobic interactions. The surface charges (generating electrostatic repulsions) and excluded volume (generating steric hindrance) of the macromolecule are proportional to *r*^2^ and *r*^3^, respectively, whereas the contact surfaces or intermolecular hydrophobic interactions remain constant, regardless to the size change of macromolecule. Thus, the surface charges and excluded volume of soluble macromolecules could be important stabilizing factors.

Mechanistically, the aggregation inhibition by the charge and steric factors of macromolecules is fundamentally different from that by hydrophobic interactions, although they are not mutually exclusive. In particular, both charge and steric factors can inhibit aggregation without direct contact with aggregation-prone regions in the linkage context [6,47,52]. To highlight the unique features of these two factors of macromolecules with respect to aggregation, we here use simple illustrations, which are depicted in Figure 3. Let us take an example that a large soluble macromolecule (blue-colored) with a constant surface charge density is covalently linked to aggregation-prone polypeptide (red-colored), as shown in Figure 3A,B. In addition, neither conformational changes nor intermolecular interactions are assumed to exist between them. This situation can mimic the ribosome (or membrane surface)-linked nascent chains. In terms of conformational changes and hydrophobic interactions, the polypeptide, either alone or linked to macromolecule, can be thought to be same or similar with respect to folding and even aggregation. Since protein folding is generally an intramolecular reaction, the thermodynamic stability and folding (or unfolding) kinetics of the polypeptide can be little affected by the presence of the linked macromolecule. As for aggregation, however, the local surfaces of polypeptide in close proximity to the linked macromolecule could be protected from intermolecular association, due to the direct steric masking of the macromolecule (Figure 3A), as pointed out previously [47].

Aggregation is a multimolecular reaction, neither unimolecular nor bimolecular. In a multimolecular system as shown in Figure 3B, it becomes more obvious that the total excluded volume of the macromolecule can directly or indirectly inhibit the aggregation of the whole regions of the polypeptide. Likewise, the total surface charges of the macromolecule are also expected to inhibit the aggregation of the whole regions of the polypeptide in a multimolecular reaction. Using either unimolecular or bimolecular system, however, it is difficult to explain these long-range effects of the surface charges and excluded volume of the macromolecules that can be exerted without direct interactions with aggregation-prone regions and induced conformational changes.

It is a challenging task to quantitatively determine the contribution of stabilizing factors of macromolecules. A representative chaperone DnaK binds to only tiny hydrophobic regions (e.g., NR**LLL**TG) of its substrates [106]. The hydrophobic interactions for the substrate recognition have been widely believed to be mainly responsible for stabilizing the bound substrate proteins against aggregation [2,3]. However, this important assumption underlying the action mechanisms of molecular chaperones is not well demonstrated experimentally. We constructed the fusion proteins in which DnaK is linked to the *N*-termini of aggregation-prone proteins to mimic the DnaK-substrate complex and expressed them in the *E. coli* cytosol [107]. In the fusion context, the residue or domain of DnaK crucial for its substrate recognition can be changed or deleted while maintaining the complex. Neither mutation of the crucial residue nor deletion of the whole substrate-binding domain has appreciable effect on the solubilizing ability of DnaK. These findings suggest that DnaK could have its intrinsic ability to stabilize its bound substrate proteins, independent of its hydrophobic interactions with substrate proteins [107].

To further elaborate on the stabilizing factors of macromolecules including DnaK, a simplified model is presented where the radius (*r*) of macromolecule is changed while the macromolecule and polypeptide are non-covalently linked to each other via limited and constant hydrophobic interactions (Figure 3C). This model is modified from the previously reported [107]. It is likely that the replacement of the covalent linkage into the hydrophobic interactions between them little affects the stabilizing effects of intrinsic factors of macromolecule in the complex state. Given a radius (*r*) of macromolecule, its surface charges and excluded volume are proportional to *r*^2^ and *r*^3^, respectively, whereas the contact surfaces or intermolecular hydrophobic interactions remain constant, regardless to the change of macromolecule size. Thus, it was proposed that the surface charges and excluded volume of soluble macromolecules including molecular chaperones could serve as dominant and universal stabilizing factors for their linked proteins [6,107]. From this viewpoint, ribosomes and membranes appear to be ideal macromolecules for stabilizing their linked polypeptides. Consistent with the present model, the electrostatic and steric repulsions have been known to be mainly responsible for stabilizing colloids against aggregation [108]. In addition, the charged patches of hsp90 were reported to be important for the hsp90-mediated aggregation inhibition [109].

### 5.3. Significance of the Charge and Steric Factors of Macromolecules in Chaperoning Functions

The charge and steric factors of macromolecules would make a profound impact on our understanding of *de novo* protein folding *in vivo*. First, both factors well explains why soluble macromolecules, including ribosomes, lipid bilayers, and folded domains, can stabilize their linked polypeptides against aggregation, regardless of the nature of linkage type between them. Second, the hydrophobic interaction-mediated substrate recognition is not a prerequisite for chaperoning function. Third, the chaperoning activity mediated by both factors can be delivered to long range: direct interactions with aggregation-prone regions of substrate proteins are not mandatory for chaperoning function. Fourth, these factors were suggested to have a tendency to inhibit most, if not all, types of multimolecular assemblies, such as amorphous aggregation, native or non-native oligomers, and amyloid fibrils [52]. Finally, these factors are compatible with hydrophobic interactions.

## 6. Conclusions and Perspectives

The elucidation of the roles of the macromolecules, including ribosomes, lipid bilayers, and folded domains, in the aggregation of their linked nascent polypeptide chains is crucial for our understanding of *de novo* protein folding *in vivo*. Accumulating evidence clearly shows that all these macromolecules can promote the solubility and folding of their linked heterologous proteins. Nevertheless, little attention is paid to the roles of these macromolecules in *de novo* folding of endogenous proteins. Thus, the known chaperoning function of these macromolecules in the linkage context needs to be incorporated into the mechanisms of *de novo* folding of endogenous proteins. In addition, we suggest that the surface charges and excluded volume of soluble macromolecules could be important and universal factors for stabilizing their linked proteins against aggregation.

After the release from ribosomes or lipid bilayers, proteins interact with a variety of macromolecules. The charge and steric factors described in this review imply that these macromolecules might have the inherent ability to exert chaperoning function on their associated proteins. Thus, this new view would contribute to a better understanding of *de novo* protein folding, aggregation-associated diseases, and proteostasis.
